# Estimating the Prevalence of Fetal Alcohol Spectrum Disorder in Australia

**DOI:** 10.1111/dar.14082

**Published:** 2025-06-02

**Authors:** Tracey W. Tsang, Daniel H. Rosenblatt, Indra Parta, Elizabeth J. Elliott

**Affiliations:** ^1^ Specialty of Child and Adolescent Health, Faculty of Medicine and Health The University of Sydney Sydney Australia; ^2^ Australian Paediatric Surveillance Unit, FASD Australian Registry, and Kids Research Sydney Children's Hospitals Network Sydney Australia; ^3^ Health Economics and Research Division Australian Government Department of Health and Aged Care Canberra Australia

**Keywords:** Australia, child, fetal alcohol spectrum disorder, prevalence

## Abstract

**Introduction:**

Fetal alcohol spectrum disorder (FASD) is caused by prenatal alcohol exposure (PAE) and characterised by severe neurodevelopmental impairment. Australian studies have reported PAE prevalence of between 14% and 78% of births. Estimating national FASD prevalence in the general population using gold‐standard active case ascertainment is costly and time‐consuming, and alternative approaches are required.

**Methods:**

Using a published equation for the risk of FASD following PAE (estimated from an international meta‐analysis) and a pooled estimate of PAE prevalence in Australia (from a meta‐analysis of 78 studies reporting 16 large general population‐based birth cohorts between 1975 and 2018), we estimated the population prevalence of FASD. Monte Carlo simulations were used to determine confidence intervals.

**Results:**

Estimated FASD prevalence in the general population was 3.64% (95% confidence interval 2.91%, 4.41%).

**Discussion and Conclusions:**

The estimated FASD prevalence in the general population of Australia was comparable to that in other high‐income countries (e.g., USA, Canada). Although it is likely that certain vulnerable populations have significantly higher FASD prevalence, this estimate provides a baseline estimate for the general population to inform service development and strategies for prevention of FASD and guide future research.

**Summary**

- Using Australian meta‐analysis data on prenatal alcohol exposure (PAE) prevalence and an international estimate of fetal alcohol spectrum disorder (FASD) risk, the prevalence of FASD in the Australian general population is estimated to be 3.64% (95% confidence interval 2.91%, 4.41%).
- The prevalence of FASD is expected to be higher in certain vulnerable Australian populations (e.g., in youth detention, foster care and remote Indigenous communities).
- Although this method has limitations, we provide the first estimate of FASD prevalence in the general Australian population, based on published Australian data on PAE prevalence and international meta‐analysis data on the risk of FASD following PAE.

## 1 Introduction

Fetal alcohol spectrum disorder (FASD) is a lifelong condition caused by prenatal alcohol exposure (PAE). It is characterised by central nervous system injury and severe neurodevelopmental impairment, with or without birth defects and associated with numerous comorbidities and substantial economic costs [1].

PAE prevalence varies widely: global prevalence is estimated at 9.8% but ranges from 0% in Oman, United Arab Emirates, Saudi Arabia, Qatar and Kuwait, to 60.4% in Ireland [2]. The global estimate of FASD prevalence in the general population of children and youth is 7.7 per 1000 population. FASD prevalence reflects the distribution of PAE, varies by World Health Organization region and country, and is significantly higher in certain vulnerable populations [3]. A meta‐analysis of 24 unique studies encompassing 1416 children estimated that one in 13 pregnancies exposed to alcohol result in a child with FASD [4].

Pooled data from a recent meta‐analysis indicate that 48% (95% confidence interval [CI] 38%, 57%) of Australian pregnancies had PAE [5]. No robust FASD prevalence data are available in general population samples, and existing data from clinic‐based studies, passive surveillance and medical record reviews are subject to under‐reporting, biased sampling and recall bias [6, 7, 8, 9], and often include small vulnerable populations [10, 11]. In a meta‐analysis of international data, the estimated FASD prevalence in Australia was 0.6 (95% CI 0, 2.8) per 1000 population [4], but the two Australian articles from which this estimate was derived included data collected over 20 years ago or from a high‐risk population not reflective of the general population.

In response to recommendations in the National FASD Strategic Action Plan (2018–2028), we aim to estimate FASD prevalence in the Australian population to inform policies and programmes [12] and overcome limitations of existing data [13]. The optimal method for estimating FASD prevalence is active case ascertainment in a representative population‐based sample, however this is expensive and time‐consuming, requires a skilled multidisciplinary clinical team, and presents ethical challenges when seeking consent for participation.

Lange et al. proposed a formula to estimate FASD prevalence when empirical data are unavailable, using the quotient of the average number of alcohol‐exposed pregnancies that would result in one case of FASD (one in 13) [4]. Using this approach, and incorporating self‐reported PAE data, Romeo et al. estimated FASD prevalence in the general New Zealand population [14]. The aim of our study is to estimate FASD prevalence in the Australian general population using Australian PAE data [5] and the methods of Lange et al. [4] and Romeo et al. [14].

## 2 Methods

### 2.1 FASD Prevalence

FASD prevalence was calculated using estimates of PAE prevalence from an Australian meta‐analysis [5], and Lange's estimate of the risk of FASD following PAE, which was based on international data [4].

$$P_{F\_\mathrm{AUS}}=\frac{P_{\text{drink}\_\mathrm{AUS}}}{N_{\text{drink}\_\text{woman}\_F}}$$

A point estimate for FASD prevalence was calculated using the equation above [14], where $P_{F\_\mathrm{AUS}}$ is the predicted prevalence of FASD in Australia, $P_{\text{drink}\_\mathrm{AUS}}$ is the estimated prevalence of PAE in Australia and $N_{\text{drink}\_\text{woman}\_F}$ is the estimated number of pregnant women who must consume alcohol for one case of FASD. Studies specifically targeting vulnerable populations were excluded from the meta‐analysis on PAE because they were not considered representative of the general population.

The Australian PAE prevalence $\left(P_{\text{drink}\_\mathrm{AUS}}\right)$ was derived from a pooled sample of 16 large birth cohorts reported in 78 studies published between 1975 and 2018 (48% (95% CI 38%, 57%)) [5]. The $N_{\text{drink}\_\text{woman}\_F}$ value was one in every 13 exposed pregnancies [4]. A 95% CI around this estimate was calculated by drawing random samples from a binomial distribution matching the FASD incidence values (given PAE) reported in these studies (a pooled sample size of 158,161) [2, 4]. This resulted in an estimated rate of FASD births among women who consumed alcohol during pregnancy of 7.69% (95% CI 7.25%, 7.90%).

A 95% CI for the resultant point estimate was derived using Monte Carlo simulation. For this purpose, $P_{\text{drink}\_\mathrm{AUS}}$ was assumed to follow a beta distribution (*α* = 50.49, *β* = 54.69) and $N_{\text{drink}\_\text{woman}\_F}$ was modelled using a binomial distribution. One million samples were drawn from each of these distributions and these values were entered into the equation, producing a full distribution for $P_{F\_\mathrm{AUS}}$ from which CIs could be attained. The 97.5th and 2.5th percentiles of the resulting distribution were used as the CI. The Monte Carlo simulations were conducted using R 4.3.1.

### 2.2 Sensitivity Analysis

A sensitivity analysis was conducted using estimates of PAE from the Australian Institute of Health and Welfare's National Drug Strategy Household Survey (NDSHS) [15, 16, 17] because these nationally administered survey data were excluded from estimates of PAE used by Young et al. from published peer‐reviewed studies [5]. The NDSHS collects information about alcohol and drug use, attitudes and perceptions and is drawn from private dwellings using stratified, multistage random sampling. Table 10.22 in the NDSHS contains information on the quantity and frequency of alcohol consumption by pregnant women aged 14–49, which is used to estimate PAE. The NDSHS, conducted every 3 years since 1985, allows estimation of PAE and FASD prevalence over time. Estimated PAE prevalence from the 2019 (29.7% (95% CI 25.3%, 34.1%)) [15] and 2022–2023 (28.3% (95% CI 21.2%, 35.4%)) NDSHS [16] were used to estimate FASD prevalence for the sensitivity analysis.

## 3 Results

### 3.1 Prevalence of FASD in Australia

Lange et al. estimated that one in every 13 women who consume alcohol during pregnancy, or 7.69% (95% CI 7.25%, 7.9%), would give birth to a child with FASD [4]. Using a pooled sample of 78 studies encompassing 16 separate Australian birth cohorts, Young et al. estimated PAE prevalence in Australia to be 48% (95% CI 38%, 57%; range: 14.2%, 76%) [5]. By incorporating these figures into the equation, the prevalence of FASD in Australia was estimated as 3.64% (95% CI 2.91%, 4.41%; range: 1.09%, 5.84%).

### 3.2 Sensitivity Analysis

Based on the PAE prevalence reported in the latest two Australian NDSHS, the estimated prevalence of FASD was 2.25% (95% CI 1.91%, 2.63%) in 2019 [15] and 2.15% (95% CI 1.62%, 2.75%) in 2022–23 [16]. Based on the CIs, the difference between these values was not statistically significant.

## 4 Discussion and Conclusions

### 4.1 FASD Prevalence in Australia

FASD prevalence in the Australian general population was estimated at 3.6% (95% CI 2.9%, 4.4%) using best available data on PAE in Australia from a systematic review with meta‐analysis. This equates to 36 (95% CI 29, 44) cases of FASD per 1000 children or 10,331 children born with FASD in 2023.

Our FASD prevalence estimate is comparable to estimates in other high‐income countries including the United States (3.11% (95% CI 1.61%, 5.40%) to 9.85% (95% CI 5.75%, 13.95%)) and Canada (1.81 (95% CI 1.08%, 2.63%) to 2.93% (95% CI 1.24%, 5.62%)) [18, 19]. In New Zealand, based on the method used in this study, the estimated FASD prevalence was lower than in Australia, at 1.7% (95% CI 1.0%, 2.7%) in 2012/2013 and 1.3% (95% CI 0.9%, 1.9%) in 2018/2019 [14]. At both time points, prevalence was significantly higher in Māori than in Pasifika and Asian populations, but not in European/other populations. FASD prevalence is likely significantly higher in certain vulnerable Australian groups than our estimate in the general population, as indicated by studies in juvenile justice detention (36%) and remote Aboriginal communities (19%), but will vary across sub‐groups and regions [10, 11].

A sensitivity analysis, performed for comparison using nationally administered survey data (NDSHS) [15, 16, 17] that was not included in Young's systematic review, showed no significant change in reported PAE (30% and 28%) or estimated FASD prevalence (2.25% and 2.15%) between 2019 and 2022–23. However, the rate of risky drinking among females of childbearing age (18–24 years) increased by 5% within the same period [17], a trend which may impact future FASD prevalence. A limitation of NDSHS data on PAE is that it is based on self‐report and not empirically verified using external screening measures. Also, the response rate for the 2022–23 survey was only 43.9% and there is likely under‐reporting, thus, estimates of PAE and FASD using these data are likely underestimates. Our study underscores the need to collect more comprehensive, contemporary and accurate national data on substance use in pregnancy.

### 4.2 Significance

FASD is the most common preventable cause of neurodevelopmental disability and birth defects with lifelong adverse health outcomes and significant financial implications [1]. In Canada, the estimated direct and indirect costs of FASD in 2013 were 1.28 and 2.34 billion CAD, respectively [20]. This includes direct costs associated with healthcare, screening, diagnosis, prescription drugs, education, community services, justice, prevention and research and indirect costs due to productivity losses for people with FASD. Prevalence data from our study will allow for preliminary estimates of the costs of FASD in Australia to inform policy and resource allocation.

We have responded to the National FASD Strategic Action Plan for 2018–28, which notes that without FASD prevalence data in the general Australian population the scale of its economic and social impacts cannot be quantified to inform policymakers or advocate for clinical services. The absence of prevalence data also has downstream repercussions, such as the exclusion of FASD from recent research estimating the total cost of alcohol to Australian society and the need for government action [21].

### 4.3 Limitations

The method used to estimate FASD prevalence has limitations. First, the risk of FASD is based on estimates subject to potentially biased or inconsistent/incomplete assessment and diagnosis of FASD. Second, there are inherent inaccuracies in PAE data, for example, recall bias, under‐reporting, stigma and difficulty measuring the quantity, frequency or timing of PAE. These factors may result in underestimation of PAE (and therefore FASD) prevalence. Furthermore, the meta‐analytic estimate of PAE prevalence used in this study is based on data from studies published between 1975 and 2018. Thus, our approach does not provide a contemporary point‐in‐time estimate of FASD prevalence and may obscure upwards trends in alcohol use in pregnancy. Nevertheless, published data on which estimates were based were robust, Australian, and derived from large birth cohorts. Compared with active case ascertainment, the method we used to estimate prevalence was time‐ and cost‐effective and provides a baseline value to inform policymakers.

### 4.4 Implications

Our FASD prevalence estimate of nearly four per 100 live births in Australia is consistent with estimates from similar populations internationally and has significant implications for health, disability, justice, community and educational sectors. It highlights the need for an increase in accessible, public, multidisciplinary diagnostic and support services, including early intervention, to improve health and wellbeing for individuals living with FASD and their families. Services for children are currently limited, with long wait times, and there is little national capacity for addressing FASD in adults. The data strongly support the urgent need for effective strategies to prevent harm from PAE.

## Author Contributions

Each author certifies that their contribution to this work meets the standards of the International Committee of Medical Journal Editors.

## Conflicts of Interest

The authors declare no conflicts of interest.

## Data Availability

The data that support the findings of this study are available from the corresponding author upon reasonable request.
